# Loxapine in the Treatment of Manic and Psychotic Symptoms in an Individual Intolerant to Multiple Mood-Stabilizing and Antipsychotic Medications

**DOI:** 10.1155/2023/8887553

**Published:** 2023-06-10

**Authors:** David S. Im, Marina Capitanov, Amy M. VandenBerg

**Affiliations:** ^1^Department of Psychiatry, University of Michigan Medical School, USA; ^2^University of Michigan Health, USA; ^3^University of Michigan College of Pharmacy, USA

## Abstract

First-line treatments for schizophrenia and schizoaffective disorder include antipsychotics and mood stabilizers, but their use may at times be limited due to severe adverse events. This case describes a 41-year-old male with a history of schizoaffective disorder and polysubstance use who was admitted to an inpatient psychiatry unit for acute manic and psychotic symptoms in the setting of absconding from his residential home and noncompliance with prescribed psychiatric medications. During his inpatient psychiatric hospitalization, he experienced DRESS (drug reaction with eosinophilia and systemic symptoms) with valproate, nephrogenic diabetes insipidus with lithium, potential neuroleptic malignant syndrome with risperidone, and orthostasis/tachycardia with clozapine. He ultimately achieved stabilization of manic and psychotic symptoms with loxapine without experiencing adverse events. This report highlights the potential utility of loxapine in individuals with schizoaffective disorder intolerant to standard mood-stabilizing and antipsychotic medications.

## 1. Introduction

Treatment of schizophrenia and schizoaffective disorder poses many challenges from potential adverse effects of medications to limited options for treatment refractory symptoms. Although second-generation antipsychotics have a lower risk of extrapyramidal symptoms (EPS), tardive dyskinesia, and neuroleptic malignant syndrome (NMS), these serious adverse effects still occur. Clozapine remains the gold standard treatment for refractory symptoms, with antipsychotic polypharmacy and other augmentation strategies, including mood stabilizers, as second-line options [1]. These agents carry their own risks, from thrombocytopenia and DRESS (drug reaction with eosinophilia and systemic symptoms) with valproate [2, 3] to diabetes insipidus and a narrow therapeutic index for lithium [4, 5]. Carbamazepine and oxcarbazepine both increase the risk of hyponatremia and rare blood dyscrasias and potentially interact with most second-generation antipsychotics to decrease effectiveness via CYP3A4 and P-glycoprotein induction [6, 7].

In addition to class-based adverse effects, clozapine carries the known risk of neutropenia and the unique risks of cardiac toxicity, tachycardia, and gastrointestinal hypomotility, which may limit its use. Loxapine is a mid-potency first-generation antipsychotic with key similarities and differences compared to clozapine. Clozapine and loxapine are nearly identical in structure and have similar high-potency binding as 5-hydroxytryptamine 2A (5-HT_2A_) receptor antagonists. Loxapine is a more potent dopamine type 2 (D_2_) receptor antagonist but has a much lower affinity for alpha-adrenergic type 2 (alpha-2), histamine, and muscarinic receptors [8]. This profile results in an EPS risk similar to olanzapine but a much lower risk of orthostasis, sedation, tachycardia, and gastrointestinal hypomotility compared to clozapine. Additionally, loxapine is not associated with a neutropenia risk. The lack of well-controlled studies on its use in treatment-refractory schizophrenia remains a limitation of loxapine [9, 10].

We present the case of a patient with treatment-refractory schizoaffective disorder who was admitted to our inpatient psychiatry unit for severe manic and psychotic symptoms. The patient initially responded well to risperidone, then proceeded to experience a series of serious adverse effects with this medication, clozapine, divalproex, and lithium, leading to prolonged medical hospitalization before eventually stabilizing on loxapine. This case is unique in that (1) the patient involved demonstrated a severe sensitivity to multiple standard medications used in the treatment of mania and psychosis, necessitating intensive care unit stays on two occasions, and (2) it illustrates the potential utility of loxapine in treating manic symptoms in patients intolerant to standard antimanic and antipsychotic medications. Our hospital's Institutional Review Board (IRB) does not require IRB review to publish case reports. The patient's name, dates of treatment, county of residence, and other potentially identifying information have been omitted from this report to preserve anonymity. To maximize the accuracy, transparency, and usefulness of this case report, we completed a CARES checklist, included as a Supplementary File (available here).

## 2. Case Presentation

Our patient was a 41-year-old man with diagnoses of schizoaffective disorder and polysubstance use disorder who was admitted to the inpatient psychiatry unit after an extended stay (24 days) in the psychiatric emergency department, where he presented with manic and psychotic symptoms, including mood lability, pressured speech, grandiosity, paranoia, and disorganized thinking, in the context of medication noncompliance and elopement from his group home. His prolonged psychiatric emergency department course was due to difficulty finding an available inpatient psychiatric bed at or within a reasonable radius of the receiving hospital. Per the patient's guardian, medications confirmed 6 weeks prior to admission included divalproex extended-release (ER) 500 mg twice daily, clonazepam 0.5 mg twice daily, risperidone 4 mg twice daily, risperidone microspheres long-acting injection 25 mg every 2 weeks, quetiapine 400 mg daily, and trazodone 100 mg daily. Based on the fill history and patient report, nonadherence was likely in the weeks prior to admission.

Limited information was available regarding the patient's past psychiatric history, as he was a difficult historian due to his mental state at the time of the initial assessment, as noted above. From a review of available records, he was noted to have been diagnosed with schizoaffective disorder in his early twenties and to have been prescribed multiple medications at various points to address manic and psychotic symptoms, including risperidone (up to 8 mg per day of the oral form and up to 25 mg every 14 days of the long-acting injectable form), quetiapine (up to 400 mg per day), and divalproex (up to 1500 mg per day). He was also prescribed trazodone for sleep (up to 50 mg per day) and sertraline for anxiety (unclear dose and duration per records). A note was made of at least partial response to risperidone and divalproex for manic and psychotic symptoms, though the durability of these effects was difficult to assess due to poor compliance. Records documented a history of over 30 psychiatric hospitalizations, usually in the setting of medication noncompliance leading to symptom relapse, with two such hospitalizations in the year prior to presentation. Mention was also made of a suicide attempt three years prior to the presentation, though the circumstances of this were unclear (the patient had reported this to an emergency room provider at an outside hospital; he denied any such history on evaluation in our psychiatric emergency room).

A history of “semi-regular” use of alcohol and cannabis was also noted in his records, beginning in his early twenties, though specific amounts, frequency, adverse consequences, and any treatment for substance use were not documented. Notably, his urine toxicology screen was negative at the time of presentation to our psychiatric emergency room.

Medically, a history of acquired immune deficiency syndrome (AIDS) (for which the patient had refused treatment with medications) was noted, with the last documented CD4 count being in the 430s range at an outside hospital one year prior to presentation.

Family history was significant for schizophrenia and suicide in a maternal uncle. In terms of social history, records indicated that the patient was an only child and that his parents separated when he was an infant; he was noted to have had no relationship with his father. He reported no history of domestic or family conflicts but also noted no current social support at the time of presentation. There was no documented history of his experiencing physical or sexual abuse, though he referred in a prior emergency room visit to a history of “verbal abuse” (unclear how long ago or by whom). He was also noted to have been married in the past but separated for 20 years prior to the presentation; he had no children. He was noted to have lived in numerous group homes over the prior ten years due to mental health issues, with frequent periods of homelessness between these stays, including for three days prior to presentation after he had absconded from his last group home due to paranoid concerns about the staff and residents there. Educationally, he was noted to have completed high school and college but to have been unemployed for ten years prior to presentation, receiving disability income, and having a guardian appointed to him due to mental health issues.

Physical examination upon initial presentation in the psychiatric emergency room was unremarkable other than the patient appearing disheveled, irritable, and guarded. Vital signs were within normal limits. Laboratory tests, including electrolytes, liver function tests, complete blood count, thyroid function tests, urine analysis, and urine drug screen, were all within normal limits.

On mental status examination, he showed loud, pressured speech, guarded and labile affect, irritable and elevated mood, disorganized (tangential, loose) thinking, paranoid delusions that staff and residents at the group home where he had been living were involved in a conspiracy against him, no reported suicidal or homicidal ideation, and poor insight (e.g., denying that he had any mental health issues and that he only needed sertraline to “clear his head”). The diagnostic impression at the time of admission to our inpatient psychiatry unit was schizoaffective disorder, bipolar type. He was assessed to have a Young Mania Rating Scale (YMRS) [11] score of 50 at the time of admission.

During his psychiatric emergency department stay and for the first 19 days of admission, the patient refused all medications, as allowed under the Michigan Mental Health Code. Following a court hearing on hospital day (HD) 20, he was court-ordered to receive medication treatment, which initially included oral risperidone (titrated over eight days to 6 mg at bedtime) and divalproex ER (titrated over ten days to 2000 mg at bedtime, approximately 24.5 mg/kg per the patient's weight of 81.6 kg), with a 13-hour serum valproic acid level of 82.8 mcg/mL on HD 37. Additionally, lithium was started on HD 28 and titrated over three days to 900 mg orally at bedtime with a 13-hour serum level of 0.80 mmol/L on HD 33. Lorazepam, 1 mg orally three times daily, was also prescribed to address anxiety. He demonstrated a gradual improvement in psychotic and manic symptoms on this regimen over the course of three weeks, to the point of approaching discharge from the hospital on HD 52, as reflected by a reduction in his YMRS score to 13.

On HD 53, he presented with fever (temperature maximum 101.1 degrees Fahrenheit), tachycardia (heart rate maximum 136 beats per minute), and hypotension (blood pressure minimum 80 systolic over 53 diastolic). Laboratory tests revealed leukocytosis (white blood cell count 10,800/mcL) and an elevated serum lactate level (3.4 mmol/L). He was transferred to the critical care medical unit (CCMU), where an infectious workup was negative, but a diffuse, erythematous, polymorphous rash on his face, neck, arms, and chest was noted on HD 54. The rash, fever, tachycardia, hypotension, and eosinophils of 900/mcL on HD 57 (increasing over the next six days to 4800/mcL) were consistent with DRESS, with divalproex the most likely cause. Divalproex was discontinued on HD 57, and the patient was treated with hydrocortisone 1% cream, a one-time dose of intramuscular triamcinolone 80 mg, and a four-week oral prednisone taper. Lithium (immediate release) was increased from 900 mg to 1200 mg at bedtime for mood stability after divalproex discontinuation. On HD 64, after 6 doses of 1200 mg, the 24-hour serum lithium level was 1 mmol/L. Although the patient refused a skin examination, he reported subjective improvement in the rash.

On HD 61, he was transferred back to the inpatient psychiatry unit, where he was noted to be irritable, grandiose, and paranoid on a regimen of risperidone 6 mg at bedtime, lithium 1200 mg at bedtime, and oral lorazepam 1 mg three times daily. His YMRS score at that point had increased to 44. On HD 63, he presented again with tachycardia, fever, leukocytosis, and hypotension, prompting a return to the CCMU, where further laboratory tests revealed lactic acidosis (serum lactate 5.3 mmol/L), acute kidney injury (serum creatinine 1.57 mg/dL, estimated glomerular filtration rate 56 mL/min/1.73 m^2^), and an elevated serum creatine phosphokinase (CPK) of 2206 units/L. He was treated with vasopressors and broad-spectrum antibiotics for distributive shock, with the etiology ultimately determined to be DRESS (biopsy confirmed) due to divalproex, with a potential NMS component. On HD 63, risperidone was increased to 7 mg at bedtime but ultimately discontinued on HD 64 due to suspicion of NMS.

On HD 66, polyuria with hypernatremia (serum sodium of 147 mmol/L, an increase from 131 mmol/L drawn 17 hours prior and increasing to 150 mmol/L over the next 24 hours) developed, and lithium was discontinued. Following an unremarkable workup for central nervous system causes (including magnetic resonance imaging of the brain) and resolution of polyuria and hypernatremia, symptoms were attributed to lithium-induced nephrogenic diabetes insipidus, for which he was treated with subcutaneous desmopressin.

On HD 68 and 69, respectively, the patient evidenced two episodes of differing arrhythmia for which the cardiology and electrophysiology teams were consulted. The first arrhythmia was a wide-complex rhythm thought to be benign. The second arrhythmia was a 1- to 2-minute episode of sustained ventricular tachycardia that resolved with suctioning. Loading doses of intravenous amiodarone were given and transitioned to oral amiodarone. Bictegravir/emtricitabine/tenofovir alafenamide was also initiated for the treatment of acquired immune deficiency syndrome (AIDS) at the recommendation of the infectious diseases team.

With the discontinuation of divalproex due to DRESS, antipsychotics due to potential NMS, and lithium due to nephrogenic diabetes insipidus, manic and psychotic symptoms intensified (e.g., the patient was observed singly loudly at night, making sexual overtures toward female staff, asserting grandiose occupational beliefs, and distrusting staff), with a corresponding increase in his YMRS score to 49. On HD 69, clozapine was initiated due to its lower risk of NMS. The absolute neutrophil count was 4300/mcL immediately prior to the first dose of clozapine. Low blood pressure (94 to 106 systolic/52 to 63 diastolic) and heart rate lability (69 to 147 beats per minute) despite adequate oral intake and hydration prevented titration above 25 mg twice daily. Clozapine was discontinued and switched to loxapine, 10 mg at bedtime on HD 80, and titrated to 20 mg twice daily over the next seven days.

On HD 89, the patient was transferred back to the inpatient psychiatry unit, where he presented as irritable, disorganized, grandiose, hypersexual, and paranoid (YMRS score still 49). With close monitoring for signs of NMS, loxapine was gradually increased over the next several weeks to a dose of 50 mg in the morning and 75 mg at bedtime. He tolerated this well and demonstrated a significant reduction in the above symptoms, with his YMRS score decreasing to 15. His blood pressure remained stable in the 110s to 130s systolic/60s to 80s diastolic range, and his heart rate was in the 80s to 110s range while consistently refusing amiodarone.

He maintained consistent compliance with loxapine and lorazepam during the last several weeks of his admission, as documented in nursing medication administration records. At the time of discharge, he presented with appropriate grooming, euthymic mood, appropriate and reactive affect, nonpressured speech, organized thought processes, and no expressed paranoid ideas. His YMRS score was 8. He continued to tolerate his psychiatric medications well and reported no adverse effects. His vital signs were within normal limits (blood pressure 132/62, pulse 89, temperature 97.8 degrees Fahrenheit). Arrangements were made to have him discharged to an adult foster care home in his county of residence and to follow up with his local community mental health agency. Unfortunately, despite diligent efforts, we were unable to successfully make contact with the patient or his guardian in the 6 months following his discharge from our unit. That stated, we found no indication in our patient's medical record that he was evaluated in our psychiatric emergency room or hospitalized psychiatrically within the 6 months following his discharge.

**Figure 1:**
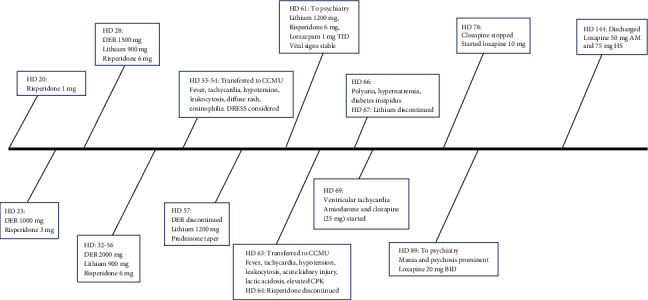
Timeline of hospital course and medication changes. HD: hospital day; DER: divalproex extended release.

Figure 1 depicts a timeline of our patient's hospital course, including medication interventions and changes.

## 3. Discussion

It is noteworthy that our patient spent 24 days in the psychiatric emergency department prior to his admission to the inpatient psychiatry unit, during which time he was allowed to refuse psychiatric medications except in emergency circumstances. This is clearly not an ideal situation for a patient with acute manic and psychotic symptoms, however, reflects statutory provisions in Michigan designed to protect the rights of individuals with mental health issues. In Michigan, the state mental health code specifies that in order for an individual to be treated on a scheduled/nonemergent basis with psychiatric medication involuntarily, a court hearing must be held in which it is determined that the individual requires mental health treatment based on having a statutorily defined mental illness and that, as a result of that illness, there is a substantial likelihood that the individual may intentionally or unintentionally harm himself/herself or another person [12]. Such court hearings are scheduled in response to the filing of a petition (typically completed by a hospital social worker) and two clinical certificates (typically completed by evaluating or admitting psychiatrists) within 24 hours of a patient's admission to a psychiatric unit [12]. Therefore, if an individual is waiting to be admitted to a psychiatric unit in an emergency setting, that individual has no obligation to accept prescribed psychiatric medication (except in emergency circumstances). This, along with difficulty finding an available inpatient psychiatric bed within a reasonable radius of the receiving hospital, contributed to an extensive interval of our patient not receiving treatment for his manic and psychotic symptoms prior to his civil commitment hearing on day 20 of his admission, increasing risks for poorer treatment response [13], longer post-hearing lengths of stay [14], and increased costs [15], a system issue not unique to our institution [13–15]. In this particular case, the patient ultimately had a favorable psychiatric outcome, though his length of stay was prolonged due to the development of medical complications (Table 1).

While the medical complications in this case were not clearly foreseeable, two risk factors for the development of NMS consistently noted in the literature—a high neuroleptic dose and a rapid rate of neuroleptic titration [16–19]—may have been applicable here. Our patient was prescribed risperidone at an ultimate dose of 6 mg/day, a high (but within acceptable therapeutic range) dose of this medication, with titration from 1 mg/day to 6 mg/day occurring over eight days (a relatively brisk titration, although within established guidelines for risperidone titration). It is possible that the acuity of the patient's presentation (including irritability, paranoia, and disorganization) and its resulting impact on the patient, staff, and therapeutic milieu may have prompted a lower threshold for near-daily adjustments in his risperidone dose, within the standard of care, but elevating his risk for NMS compared to slower titration schedules and lower ultimate doses [20]. The diagnosis of DRESS with valproate may have confounded the diagnosis of NMS. On the one hand, it may have masked the initial recognition of NMS, as both involve fever as a hallmark symptom. On the other hand, fever, agitation, and inflammation from DRESS may have led to increased serum CPK levels mimicking NMS. Due to the severity of the symptoms in this case, it was determined best to err on the side of caution and discontinue risperidone. Table 1 presents a review of the core symptoms and signs associated with each of the three adverse medical events experienced by our patient during his stay.

Although loxapine was chosen in this case because of its demonstrated lower propensity for causing orthostasis, tachycardia, and other adverse effects compared to clozapine [8], in our patient's case, the main observed vital sign difference between these two medications included a lower occurrence of hypotension with loxapine (Table 2). Our patient demonstrated largely normal heart rates on loxapine, though with some trend toward mild tachycardia (100s to 110s) at higher doses. However, his blood pressure was consistently in a more acceptable (nonhypotensive) range on this medication, and there were no indications of other adverse effects (e.g., EPS or NMS). Notably, he had refused to take his amiodarone for much of the time he was prescribed loxapine, possibly leading to ongoing tachycardia despite overall clinical improvement in manic and psychotic symptoms. Moreover, increased blockade of alpha-1 adrenergic receptors at higher doses of loxapine could account for some increase in heart rate in our patient's case.

One might question whether the ultimate reduction in manic and psychotic symptoms in our patient could have been due to the effects of antipsychotics prescribed to him prior to loxapine (specifically, clozapine, oral risperidone, risperidone microspheres, and quetiapine). Regarding the potential role of clozapine (or its active metabolite, norclozapine), studies have shown that the mean volume of distribution of clozapine at steady state ranges from 5 to 7 liters per kilogram and that its total clearance ranges from 37 to 57 liters per hour [21]. Given that our patient weighed 83 kilograms, we can estimate the volume of distribution of clozapine in his case to be approximately 498 liters. If we conservatively assume a total clearance of 37 liters per hour, it would take approximately 13.4 hours for clozapine to be eliminated from his system. This is in line with the mean terminal elimination rate of clozapine reported in the literature (elimination half-life of 10 to 16 hours) [21]. Since our patient was off clozapine and taking loxapine for 60 days before starting to experience a significant reduction in his YMRS scores, it is unlikely clozapine or its metabolite norclozapine (with a similar clearance and elimination half-life to clozapine) would have contributed to this ultimate reduction.

Similar considerations can be applied when examining the potential contributions of oral risperidone, risperidone microspheres long-acting injection, and quetiapine to the reduction in our patient's YMRS scores by HD 139. For example, the mean elimination half-lives of oral risperidone (3 to 24 hours) [22], its major metabolite 9-hydroxy-risperidone (20 to 23 hours) [22], risperidone microspheres (26 days) [23], quetiapine (5 to 7 hours) [24, 25], and norquetiapine (the main active metabolite of quetiapine, 11 to 12 hours) [24] make it improbable that any of these medications or metabolites—unprescribed to our patient for at least 71 days prior to a significant reduction in his YMRS score—played significant roles in his ultimate clinical improvement.

The onset of a significant reduction in YMRS score by day 60 of loxapine use at a dose of 125 milligrams per day is consistent with the known pharmacology of loxapine in that, while balanced 5HT_2_ and D_2_ receptor blockade is achieved at doses of less than 100 mg daily, higher doses are associated with further D_2_ receptor antagonism by active metabolites, along with greater alpha-1 adrenergic blockade and muscarinic effects, providing potential benefit for treatment-refractory individuals with psychosis [26].

Table 3 presents the YMRS scores by day and dose of loxapine prescribed.

The strengths of this report include (1) its careful documentation of medication interventions, including dosages, timelines, tolerability, and observed response (assessed by a validated scale, the YMRS [11]) for the patient in question, allowing preliminary (although not definitive) inferences to be made regarding the therapeutic or adverse impacts of interventions, (2) its meticulous description of the medical issues that developed in this case, to educate psychiatric and medical providers about the potential emergence of these issues with standard, evidence-based pharmacologic interventions for patients with schizoaffective disorder, (3) its realistic conveyance of the practical, system-based challenges in securing needed mental health treatment for individuals with acute mental health symptoms in a timely manner (and the potential consequences of delays in this regard), and (4) its illustration of the potential usefulness of an older, medium-potency, first generation antipsychotic—loxapine—in treating manic and psychotic symptoms in individuals intolerant to standard first-line medications for these symptoms, with provision of plausible explanations for its beneficial effect from a tolerability and efficacy standpoint.

Limitations of this report include the noncontrolled, nonrandomized nature of the pharmacologic interventions, making definitive conclusions about the relative efficacy and tolerability of loxapine compared to mood stabilizers and other antipsychotics in treating manic and psychotic symptoms in treatment refractory schizoaffective disorder difficult. Another limitation, as noted above, is that although the diagnosis of DRESS was confirmed by biopsy, the diagnosis of NMS may have been partly confounded by the presence of DRESS, as some symptoms (e.g., fever and inflammation) are shared between these two conditions. However, the presence of autonomic dysfunction and fever seven days after discontinuation of divalproex and in the context of recent antipsychotic use supports the development of NMS in addition to DRESS.

## 4. Conclusions

The case presented illustrates the potential utility of loxapine, a mid-potency first-generation antipsychotic with key similarities and differences compared to clozapine, in the treatment of manic and psychotic symptoms in individuals with schizoaffective disorder. The favorable clinical outcome with loxapine in this case may have been due to its relatively greater potency as a D_2_ receptor antagonist (compared to clozapine), lower propensity for causing orthostasis (compared to clozapine), and lower risk of EPS and possibly NMS (compared to other antipsychotics). Randomized, double-blind, placebo-controlled, and head-to-head comparison studies are needed to more clearly ascertain the relative efficacy of loxapine in this regard.

## Figures and Tables

**Table 1:** Adverse event review of symptoms.

Condition	Core symptoms/signs
Drug related eosinophilia and systemic symptoms (DRESS) [2, 3]	(i) Fever (38 to 40 degrees Celsius) [3] (ii) Skin rash (diffuse, polymorphic) [3] (iii) Liver injury [3] (iv) Eosinophilia [3] (v) Enlarged lymph nodes in at least 2 body sites [3]

Neuroleptic malignant syndrome (NMS) [1]	(i) Recent use of dopamine receptor antagonist (antipsychotic) [27, 28] (ii) Central nervous system (CNS) symptoms (drowsiness, mutism, confusion, and coma) [27, 28] (iii) Severe “lead pipe” muscle rigidity/other extrapyramidal effects (bradykinesia, tremor, and abnormal movements/posture) [27, 28] (iv) Hyperthermia [27, 28] (v) Autonomic dysfunction (labile blood pressure and tachycardia) [27, 28]

Diabetes insipidus [4–6]	(i) Polyuria [4, 5] (ii) Polydipsia [4, 5] (iii) Signs of dehydration (e.g., dry lips, orthostatic hypotension, weakness, dizziness, and fatigue) [4, 5] (iv) Signs of hypernatremia (serum sodium greater than 145 mEq/L), e.g., restlessness, agitation, decreased deep tendon reflexes, and seizures [4, 5]

mEq/L: milliequivalents per liter.

**Table 2:** Vital sign values with antipsychotics during our patient's admission.

Antipsychotic	Hospital day (HD)	Dose	Temperature (degrees Celsius)	Blood pressure (systolic in mmHg/diastolic in mmHg)	Heart rate (beats per minute)
Risperidone	20	1 mg QHS	36.8	136/65	117
	21	1 mg QHS	36.9	109/59	88
	22	2 mg QHS	36.8	119/77]	98
	23	3 mg QHS	37.4	108/66	109
	24	4 mg QHS	Pt refused	118/69	122
	25	4 mg QHS	Pt refused	Pt refused	Pt refused
	26	4 mg QHS	36.8	137/83	102
	27	5 mg QHS	36.7	105/71	111
	28	6 mg QHS	36.9	104/59	104
	29	6 mg QHS	36.8	110/70	107
	30	6 mg QHS	36.6	115/50	130
	31	6 mg QHS	Pt refused	102/55	126
	32	6 mg QHS	37.1	116/64	89
	33	6 mg QHS	36.7	139/57	114
	34	6 mg QHS	Pt refused	113/63	112
	35	6 mg QHS	37	116/76	88
	36	6 mg QHS	Pt refused	Pt refused	Pt refused
	37	6 mg QHS	36.6	110/55	106
	38	6 mg QHS	36.9	116/68	94
	39	6 mg QHS	36.5	104/69	112
	40	6 mg QHS	36.5	113/62	92
	41	6 mg QHS	36.9	107/60	103
	42	6 mg QHS	36.5	104/62	91
	43	6 mg QHS	36.7	117/63	103
	44	6 mg QHS	36.7	133/57	100
	45	6 mg QHS	37	112/69	79
	46	6 mg QHS	37	111/67	99
	47	6 mg QHS	36.7	112/60	89
	48	6 mg QHS	36.7	134/89	109
	49	6 mg QHS	36.5	107/57	110
	50	6 mg QHS	36.9	107/63	80
	51	6 mg QHS	36.8	102/58	89
	52	6 mg QHS	36.9	123/61	112
	53	6 mg QHS	36.9	80/53	128
	54	6 mg QHS	38.9	93/52	97
	55	6 mg QHS	39.1	96/50	110
	56	6 mg QHS	39.6	99/50	126
	57	6 mg QHS	36.9	98/52	102
	58	6 mg QHS	37.3	85/52	107
	59	6 mg QHS	37.5	92/50	105
	60	6 mg QHS	37.1	97/41	106
	61	6 mg QHS	37.4	108/41	107
	62	6 mg QHS	37.1	97/52	111
	63	6 mg QHS	39.5	90/42	121
	64	(held)	39.5	94/47	156
	65	(held)	38.3	99/57	90
	66	(held)	38.2	97/55	90
	67	(held)	38.2	89/52	82
	68	(held)	38	88/53	80

Clozapine	69	6.25 mg QHS	37.2	93/54	77
	70	12.5 mg QHS	36.5	135/90	103
	71	12.5 mg BID	37.6	107/57	96
	72	12.5 mg BID	36.6	118/60	89
	73	12.5 mg BID	36.8	103/58	86
	74	25 mg QPM	Pt refused	Pt refused	Pt refused
	75	12.5 mg QAM, 25 mg QHS	36.6	102/61	74
	76	25 mg BID	36.7	106/63	75
	77	25 mg BID	36.5	136/83	147
	78	25 mg BID	36.5	102/55	84
	79	25 mg BID	37.1	94/52	69

Loxapine	80	10 mg QHS	36.7	109/64	68
	81	10 mg QHS	36.4	107/59	80
	82	10 mg QHS	36.5	112/58	98
	83	10 mg BID	37.2	114/66	95
	84	10 mg QAM, 20 mg QHS	36.7	111/64	88
	85	10 mg QAM, 20 mg QHS	36.4	95/54	82
	86	20 mg BID	36.7	114/67	104
	87	20 mg BID	36.4	117/69	85
	88	20 mg BID	36.7	101/51	97
	89	20 mg BID	36.7	111/62	88
	90	20 mg BID	36.7	161/100	134
	91	20 mg BID	36.6	110/72	108
	92	20 mg BID	37.1	99/64	125
	93	20 mg BID	36.9	127/76	112
	94	20 mg BID	36.9	127/61	100
	95	20 mg BID	36.7	118/71	117
	96	20 mg BID	36.3	127/80	82
	97	20 mg BID	36.8	120/82	110
	98	50 mg QHS	36.5	118/85	92
	99	50 mg QHS	36.8	135/71	102
	100	50 mg QHS	36.8	122/73	64
	101	50 mg QHS	36.5	130/79	101
	102	50 mg QHS	36.6	138/90	108
	103	50 mg QHS	Pt refused	Pt refused	Pt refused
	104	50 mg QHS	37.4	133/79	97
	105	50 mg QHS	37.8	135/97	120
	106	50 mg QHS	36.8	128/94	98
	107	50 mg QHS	36.1	129/90	88
	108	50 mg QHS	36.7	144/75	100
	109	50 mg QHS	37	123/79	103
	110	50 mg QHS	37.1	114/67	93
	111	50 mg QHS	36.8	132/75	88
	112	50 mg QHS	37	109/80	99
	113	50 mg QHS	36.8	128/78	93
	114	50 mg QHS	36.4	143/69	112
	115	50 mg QHS	36.7	121/68	104
	116	50 mg QHS	Pt refused	Pt refused	Pt refused
	117	50 mg QHS	37.1	132/73	103
	118	50 mg QHS	36	142/78	101
	119	50 mg QHS	36.8	132/71	110
	120	50 mg QHS	36.5	119/68	88
	121	50 mg QHS	36.6	106/64	99
	122	50 mg QHS	36.9	123/84	108
	123	50 mg QHS	36.5	120/72	101
	124	50 mg QHS	36.6	157/78	79
	125	50 mg QHS	37.9	111/67	102
	126	50 mg QHS	Pt refused	Pt refused	Pt refused
	127	50 mg QHS	37.1	115/80	109
	128	50 mg QHS	Pt refused	118/67	106
	129	50 mg QHS	Pt refused	Pt refused	Pt refused
	130	50 mg QHS	36.1	130/71	95
	131	50 mg QHS	36.7	176/73	122
	132	25 mg QAM, 50 mg QHS	Pt refused	Pt refused	Pt refused
	133	25 mg QAM, 50 mg QHS	37.2	106/63	114
	134	50 mg BID	36.7	105/65	116
	135	50 mg BID	36.6	108/66	115
	136	50 mg BID	36.9	123/88	113
	137	50 mg BID	36.7	136/81	102
	138	50 mg BID	36.8	148/75	98
	139	50 mg QAM, 75 mg QHS	Pt refused	Pt refused	Pt refused
	140	50 mg QAM, 75 mg QHS	36.9	123/88	113
	141	50 mg QAM, 75 mg QHS	36.7	136/81	102
	142	50 mg QAM, 75 mg QHS	36.5	115/59	103
	143	50 mg QAM, 75 mg QHS	Pt refused	Pt refused	Pt refused
	144	50 mg QAM, 75 mg QHS	36	124/84	123
	145	50 mg QAM, 75 mg QHS	36.6	132/62	89

mg: milligrams; mmHg: millimeters of mercury; pt: patient; QHS: at bedtime; QAM: every morning; BID: twice daily.

**Table 3:** YMRS scores for our patient in relation to day of loxapine use.

Day of loxapine use	Dose of loxapine	YMRS score
1	10 mg QHS	49
2	10 mg QHS	49
3	10 mg QHS	49
4	10 mg BID	50
5	10 mg QAM, 20 mg QHS	49
6	10 mg QAM, 20 mg QHS	49
7	20 mg BID	48
8	20 mg BID	44
9	20 mg BID	49
10	20 mg BID	42
11	20 mg BID	40
12	20 mg BID	34
13	20 mg BID	38
14	20 mg BID	30
15	20 mg BID	26
16	20 mg BID	32
17	20 mg BID	30
18	20 mg BID	34
19	50 mg QHS	40
20	50 mg QHS	40
21	50 mg QHS	40
22	50 mg QHS	38
23	50 mg QHS	37
24	50 mg QHS	37
25	50 mg QHS	38
26	50 mg QHS	35
27	50 mg QHS	35
28	50 mg QHS	35
29	50 mg QHS	35
30	50 mg QHS	40
31	50 mg QHS	40
32	50 mg QHS	38
33	50 mg QHS	40
34	50 mg QHS	35
35	50 mg QHS	37
36	50 mg QHS	37
37	50 mg QHS	37
38	50 mg QHS	39
39	50 mg QHS	35
40	50 mg QHS	37
41	50 mg QHS	35
42	50 mg QHS	35
43	50 mg QHS	38
44	50 mg QHS	36
45	50 mg QHS	37
46	50 mg QHS	35
47	50 mg QHS	38
48	50 mg QHS	36
49	50 mg QHS	36
50	50 mg QHS	36
51	50 mg QHS	37
52	50 mg QHS	37
53	25 mg QAM, 50 mg QHS	37
54	25 mg QAM, 50 mg QHS	37
55	50 mg BID	37
56	50 mg BID	31
57	50 mg BID	32
58	50 mg BID	35
59	50 mg BID	35
60	50 mg QAM, 75 mg QHS	32
61	50 mg QAM, 75 mg QHS	28
62	50 mg QAM, 75 mg QHS	25
63	50 mg QAM, 75 mg QHS	17
64	50 mg QAM, 75 mg QHS	8
65	50 mg QAM, 75 mg QHS	8

mg: milligrams; QHS: at bedtime; QAM: every morning; BID: twice daily; YMRS: Young Mania Rating Scale.

## Data Availability

The data used to support the findings of this study are included within the article. This is a case report in which clinical information relevant to reporting of the case was obtained from the patient's medical record, with omission in the manuscript of any patient-identifying information in order to preserve patient anonymity.

## Supplementary Materials

Supplementary Materials CARE Checklist for case reports
