# Supplementary material for: Loxapine in the Treatment of Manic and Psychotic Symptoms in an Individual Intolerant to Multiple Mood-Stabilizing and Antipsychotic Medications
Source: Case Rep Psychiatry. 2023 Jun 10;2023:8887553. doi: 10.1155/2023/8887553 (PMC10276762; doi:10.1155/2023/8887553)
Supplement: Supplementary Materials — CARE Checklist for case reports [file 8887553.f1.pdf]

CARE case report guidelines

CARE Checklist of information to include when writing a case report

(cc) BY-NC-ND

Topic	Item	Checklist item description	Reported on Line
Title	1	The diagnosis or intervention of primary focus followed by the words "case report"	4,5
Key Words	2	2 to 5 key words that identify diagnoses or interventions in this case report, including "case report"	67
Abstract (no references)	3a	Introduction: What is unique about this case and what does it add to the scientific literature?	95-98
	3b	Main symptoms and/or important clinical findings	107-109; 160-166
	3c	The main diagnoses, therapeutic interventions, and outcomes	164-165; 169-175; 186; 188-189; 200-201; 221-226; 229-234
	3d	Conclusion—What is the main "take-away" lesson(s) from this case?	352-359
Introduction	4	One or two paragraphs summarizing why this case is unique (**may include references**)	95-98
Patient Information	5a	De-identified patient specific information.	99-101
	5b	Primary concerns and symptoms of the patient.	106-108
	5c	Medical, family, and psycho-social history including relevant genetic information	137-153
	5d	Relevant past interventions with outcomes	124-125
Clinical Findings	6	Describe significant physical examination (PE) and important clinical findings.	155-166
Timeline	7	Historical and current information from this episode of care organized as a timeline	See Figure 1
Diagnostic Assessment	8a	Diagnostic testing (such as PE, laboratory testing, imaging, surveys).	155-166; 205-206
	8b	Diagnostic challenges (such as access to testing, financial, or cultural)	283-287
	8c	Diagnosis (including other diagnoses considered)	164-165
	8d	Prognosis (such as staging in oncology) where applicable	Not Applicable
Therapeutic Intervention	9a	Types of therapeutic intervention (such as pharmacologic, surgical, preventive, self-care)	168-174; 186; 188-189; 200-201; 221-226; 229-231
	9b	Administration of therapeutic intervention (such as dosage, strength, duration)	169-175; 188-189; 221-226; 229-231
	9c	Changes in therapeutic intervention (with rationale)	184-186; 188-189; 200-201; 221-226
Follow-up and Outcomes	10a	Clinician and patient-assessed outcomes (if available)	236-243
	10b	Important follow-up diagnostic and other test results	231-241
	10c	Intervention adherence and tolerability (How was this assessed?)	236-237; 239-241
	10d	Adverse and unanticipated events	179-185; 194-200; 203-204; 210-213
Discussion	11a	A scientific discussion of the strengths AND limitations associated with this case report	329-349
	11b	Discussion of the relevant medical literature **with references**.	73-90; 274-283
	11c	The scientific rationale for any conclusions (including assessment of possible causes)	84-89; 354-357
	11d	The primary "take-away" lessons of this case report (without references) in a one paragraph conclusion	352-359
Patient Perspective	12	The patient should share their perspective in one to two paragraphs on the treatment(s) they received	(Pt declined)
Informed Consent	13	Did the patient give informed consent? Please provide if requested	Yes ☐ No ☒

(Potentially identifying information has been omitted from this report to preserve patient anonymity.)
